# Tooth extractions prior to chemoradiation or bioradiation are associated with weight loss during treatment for locally advanced oropharyngeal cancer

**DOI:** 10.1007/s00520-022-06942-6

**Published:** 2022-03-12

**Authors:** Doke J. M. Buurman, Anna C. H. Willemsen, Caroline M. Speksnijder, Laura W. J. Baijens, Ann Hoeben, Frank J. P. Hoebers, Peter Kessler, Annemie M. W. J. Schols

**Affiliations:** 1 Department of Cranio-Maxillofacial Surgery, Maastricht University Medical Center+, P. Debyelaan 25, 6229 HX Maastricht, The Netherlands; 2 GROW School for Oncology and Reproduction, Maastricht University Medical Center+, Universiteitssingel 40, 6229 ER Maastricht, The Netherlands; 3 Division of Medical Oncology, Department of Internal Medicine, Maastricht University Medical Center+, P. Debyelaan 25, 6229 HX Maastricht, The Netherlands; 4 Department of Respiratory Medicine, NUTRIM School of Nutrition and Translational Research in Metabolism, Maastricht University Medical Center+, Universiteitssingel 40, 6229 ER Maastricht, The Netherlands; 5 Department of Head and Neck Surgical Oncology, Utrecht University, University Medical Center Utrecht Cancer Center, Utrecht, The Netherlands; 6 Department of Oral and Maxillofacial Surgery and Special Dental Care, University Medical Center Utrecht, Utrecht University, Utrecht, The Netherlands; 7 Department of Otorhinolaryngology, Head & Neck Surgery, Maastricht University Medical Center+, P. Debyelaan 25, 6229 HX Maastricht, The Netherlands; 8 Department of Radiation Oncology, MAASTRO Clinic, Maastricht, The Netherlands

**Keywords:** Oropharyngeal cancer, Weight loss, Chemoradiotherapy, Tooth extraction, Tooth loss, Dental focal infection

## Abstract

**Purpose:**

Prior to radiotherapy combined with chemotherapy (CRT) or biotherapy (BRT) for oropharyngeal squamous cell carcinoma (OPSCC), teeth with poor prognosis that pose a risk for post-RT osteoradionecrosis (ORN) are removed. The effect of tooth loss on body weight loss and tube feeding (TF) dependency during CRT/BRT is unknown. This study aimed to evaluate the effect of incomplete dentition, tooth extractions prior to CRT/BRT, and the subsequent loss of functional units on (1) weight loss during CRT/BRT and (2) the need for TF during CRT/BRT for OPSCC.

**Methods:**

OPSCC patients treated with CRT/BRT between 2013 and 2016 were included in this retrospective cohort study. Dental status was determined during the dental assessment at first visit and after tooth extractions prior to the start of CRT/BRT. Weight loss during CRT/BRT was scored dichotomously, comparing weight loss > 5% to stable or increased weight. Potential factors associated with weight loss were identified, including patient, tumor, and treatment characteristics.

**Results:**

Seventy-seven OPSCC patients were included. Forty patients (52%) experienced weight loss > 5% during CRT/BRT. Extractions were performed in 66% of the OPSCC patients. The mean number of extracted teeth was 4.1 ± 5.6 per patient. Tooth extractions prior to CRT/BRT were associated with weight loss > 5% during CRT/BRT (HR 1.130 (95% CI 1.011–1.262), *p* = 0.031). None of the dental status-related parameters showed any significant associative value for TF during CRT/BRT.

**Conclusions:**

Pre-CRT/BRT tooth extractions intended to reduce the risk of ORN, are a risk factor for weight loss during CRT/BRT for OPSCC.

**Trial registration number:**

This study was approved by the medical ethics committee of the MUMC + (METC 2020–1589) on July 28, 2020.

## Introduction

The incidence of oropharyngeal cancer, predominantly squamous cell carcinoma, has increased over the past 30 years from less than 300 new diagnoses in the early 1990s to nearly 700 in 2018 in the Netherlands alone [1]. This is consistent with global figures, in which the increased incidence of human papilloma virus (HPV) − related oropharyngeal squamous cell carcinoma (OPSCC) has the largest share in this growth, especially among men in developed countries [2]. A better prognosis for HPV-positive OPSCC, combined with young age at diagnosis and thus a longer life expectancy, has increased awareness of late treatment-related toxicity [3]. Radiotherapy (RT) alone or in combination with chemotherapy (cisplatin) (CRT) or biotherapy (cetuximab) (BRT) is the main therapy for OPSCC with osteoradionecrosis (ORN) as one of the most feared toxicities. Although the risk of ORN has decreased with current advancements in radiotherapy techniques and better oral health regimens, cancer located in the oropharynx remains a risk factor for ORN due to its location proximate to the mandible [4–7]. Comprehensive dental assessment of potential oral sources of infection (poor prognosis teeth) prior to RT is an example of improved oral health regimes. In the Netherlands, oral health recommendations prior to RT are based on a protocol that dates from 1992, which has been revisited in 2018 [8–10]. Removal of poor prognosis teeth that are identified as a potential oral source of infection is a common recommendation in the prevention of ORN. This is however complex and controversial. Tooth extractions result in a reduced number of functional units (Table 1) and impair the ability to masticate and swallow, contributing to decreased health-related quality of life (QoL) [6, 11–13]. Indeed, this deterioration in mastication has been associated with oropharyngeal dysphagia [14, 15]. Furthermore, it has been demonstrated that oropharyngeal dysphagia is significantly related to involuntary weight loss [16, 17]. Cachexia, clinically characterized by unintended weight loss and low muscle mass [18], has a negative effect on treatment-related toxicity and oncological outcome. Head and neck cancer patients with weight loss and/or low muscle mass experienced higher levels of toxicity, more unplanned hospital admissions, and poorer overall survival [19–21]. Therefore, it is of utmost importance to prevent weight loss during oncological treatment and to elucidate contributing risk factors [21].

**Table 1:** Terminology clarification

Edentulous	No functional teeth in place
Functional tooth	A tooth was considered functional if it could make contact with an opposing (prosthetic) tooth. Roots or impacted teeth are considered as nonfunctional
Functional unit	Functional tooth, bridge pontic, or crown (on implants), which could make contact with an opposing (prosthetic) tooth, is considered a functional unit
Occlusal unit [22]	A measure to represent the chewing surface of the postcanine functional unit. One pair of occluding premolars is equal to one occlusal unit. One pair of occluding molars is considered as two occlusal units. Third molars are excluded
Eichner index [23, 24]	A validated measure describing the existing posterior functional units in support zones. It is divided into 3 main classes
Eichner index A	Functional units exist in all 4 posterior support zones
Eichner index B	Functional units are present in one to three posterior support zones or within the anterior area only
Eichner index C	No functional units left

Nutritional management targeting malnutrition to prevent or limit weight loss is an essential part of head and neck oncological treatment. Regularly, tube feeding (TF) may be necessary to achieve these goals [25].

A systematic review of longitudinal studies revealed inconsistent findings on the association between tooth loss and nutritional status in adults [26]. To our knowledge, to date, no studies have investigated the effect of incomplete dentition or loss of functional units due to tooth extraction prior to CRT/BRT, on body weight and TF dependency in patients with head and neck cancer.

Therefore, the aim of this study was to evaluate the effect of incomplete dentition, tooth extractions prior to CRT/BRT, and the subsequent loss of functional units on the following: (1) weight loss during CRT/BRT and (2) the need for TF during CRT/BRT for OPSCC. We hypothesized that OPSCC patients who underwent tooth extractions prior to RT experienced greater weight loss during CRT/BRT and were more prone to TF dependency compared to patients whose teeth were not removed.

## Materials and methods

### Study design and population

Patients with OPSCC, who were treated with primary or postoperative CRT/BRT in the Comprehensive Cancer Center of Maastricht University Medical Center (MUMC +) and Maastro Clinic between January 2013 and December 2016, were included in this retrospective cohort study. Exclusion criteria were single modality treatment with radiotherapy only, previous head and neck radiation, and TF dependency at the start of the oncological treatment. Patients were part of a larger MUMC + sample from a cohort study on alterations in body composition in locally advanced head and neck squamous cell carcinoma (LAHNSCC) [21]. Additional data extraction on dental status from the electronic health records was performed by an experienced maxillofacial prosthodontist (DB). This study was approved by the medical ethics committee of the MUMC + (METC 2020–1589).

All patients received primary CRT or BRT (cisplatin or cetuximab, respectively) or postoperative CRT (cisplatin) with curative intent. RT was administered using intensity-modulated RT (IMRT) for 5 days per week for 6 (BRT) or 7 (CRT) weeks, in fractions of 2 Gy. Cisplatin was administered intravenously in doses of 100 mg/m^2^ every 3 weeks [27, 28] concurrently with daily fractionated IMRT up to 66 Gy in 33 fractions or 70 Gy in 35 fractions in case of postoperative and primary RT, respectively. Cetuximab was indicated in patients not fit for cisplatin and consisted of a 400 mg/m^2^ loading dose, followed by 250 mg/m^2^ weekly, combined with accelerated fractionated IMRT up to 68 Gy in 34 fractions in 38 days [29]. According to the national standard procedures, the dental status was assessed through oral and radiographic examination (e.g., orthopantomography), at least 14 days before the start of CRT/BRT [8–10]. Teeth with a poor prognosis due to extensive caries, advanced periodontal disease, and non-restorable teeth were considered potential sources of infection for ORN. Radiographic abnormalities like apical radiolucency, (partially) impacted teeth, residual root tips, root resorption, and dental cysts were also considered potential sources of infection. Poor prognosis teeth within the estimated radiation fields were treated, usually by extraction.

During CRT/BRT, instructions were given to continue normal daily oral care (tooth brushing and/or interdental cleaning) as long as possible and to rinse the mouth with salt-baking soda solution 8 to 10 times a day [8, 9]. Patients received custom-made fluoride trays in combination with a neutral 1% sodium fluoride gel to be used every other day [8, 9]. To relieve the symptoms of mucositis, patients were sprayed with saline 3 times a week by the dental hygienist [30].

Patients were counselled by a dietician on a weekly basis according to the Dutch malnutrition guideline as part of standard clinical care [31]. TF was indicated if oral intake including oral nutritional supplements did not meet > 75% of the calculated nutritional requirements. TF was administered through a nasogastric tube, percutaneous endoscopic gastrostomy, or radiologically inserted gastrostomy.

### Anthropometric measurements

Weight was measured weekly at the start of RT during the standard visits to the Comprehensive Cancer Center of MUMC + . Height was measured only once before the start of CRT/BRT to calculate the body mass index (BMI). Pretreatment weight loss was a patient-reported outcome measure. Weight loss during the course of CRT/BRT was converted into a binary variable, comparing losses of more than 5% to stable or increased weight, based on the definition of grade 1 weight loss in the Common Terminology Criteria for Adverse Events Version 5.0 (CTCAE).

The same CTCAE version was also used by the radiation oncologists to report the severity of oropharyngeal dysphagia at start of RT. At the same time, the World Health Organization performance status (WHO PS) was assessed. The Charlson comorbidity index (CCI) was determined based on the medical history in the individual electronic health records [32]. The p16 status was used as a surrogate marker for HPV infection [33].

Dental status was determined at two time points: during the dental assessment at first visit (dental sources of infection and functional dental status) and after tooth extractions prior to the start of CRT/BRT (functional dental status). The dental terminology and classification systems used are listed in Table 1. Whether or not patients underwent tooth extractions, the number of extracted teeth and additional dental interventions including the removal of exostoses and implant insertion were recorded. The use of TF during CRT/BRT was treated as a binary measure, consisting of TF started during CRT/BRT for any duration versus remaining on a total oral diet.

### Statistical analyses

Descriptive statistics were reported as means and standard deviations (SDs) for normally distributed, continuous variables, and medians and interquartile ranges (IQRs) for non-normally distributed data. Comparisons between groups were performed with independent t-tests in case of a normal distribution or the Mann–Whitney *U* test in case of non-normal distribution. Normal distribution was verified using the Shapiro–Wilk test. Cross-tabulations were made for categorical variables. A chi^2^ test was used for categorical outcomes. When more than 20% of cells had expected frequencies < 5, we used Fisher’s exact test.

All potential associative variables for weight loss underwent screening through univariable logistic regression. Factors with *p* < 0.10 were selected as potentially relevant associative variables and subsequently tested using multivariable logistic regression. Due to limited sample size, the influence of potential associative factors was tested individually, with a maximum of three variables in the multivariable model.

Statistical analyses were regarded as significant if the *p* value was equal to or lower than 0.05. Data were evaluated using SPSS (IBM version 25 for Windows, Armonk, NY, USA). For the Fisher’s exact test with more than 2 by 2 items, the R software (R Core Team (2021) R Foundation for Statistical Computing, Vienna, Austria) was used.

## Results

Seventy-seven patients with OPSCC met the inclusion criteria and were included in this study. Extractions were performed in 66% of the OPSCC patients. The mean number of extracted teeth was 4.1 ± 5.6 per patient. During CRT/BRT, 40 patients (52%) experienced significant weight loss of more than 5%. Baseline characteristics are presented in Table 2. Patients with significant weight loss during CRT/BRT had a higher BMI at the start of treatment compared to patients without significant weight loss. In addition, a higher proportion of patients with significant weight loss had teeth removed to clear them from potential sources of infection.

**Table 2:** Baseline characteristics

	*Stable weight or less than 5% loss during CRT/BRT* *N* = *37 (48%)*	> *5% weight loss during CRT/BRT* *N* = *40 (52%)*	*p* Value
Patient characteristics			
Age (years)			
Mean ± SD	58.4 ± 9.5	59.4 ± 6.0	
Median (IQR)	60.0 (13)	59.5 (9)	0.971^c^
Male	25 (68%)	29 (73%)	0.637^d^
Female	12 (32%)	11 (28%)	
Smoking history	33 (89%)	35 (88%)	1.000^a^
No history of smoking	4 (11%)	5 (13%)	
Alcohol consumption	19 (51%)	27 (68%)	0.149^d^
No alcohol consumption	18 (49%)	13 (33%)	
BMI at start RT ((kg/m^2^); mean ± SD	24.5 ± 5.0	26.7 ± 4.2	**0.039**^**b**^
Percentage weight loss prior to CRT/BRT; mean ± SD	2.4 ± 3.7	1.7 ± 3.2	0.373^b^
Dysphagia (CTCAE grade)			
0—No symptoms of dysphagia	18 (49%)	15 (38%)	0.077^d^
1—Symptomatic, regular diet	7 (19%)	17 (43%)	
2—Symptomatic, altered eating/swallowing	12 (32%)	8 (20%)	
WHO PS 0	9 (24%)	14 (35%)	0.325^a^
WHO PS 1	28 (76%)	25 (63%)	
WHO PS 2	0 (0%)	1 (3%)	
CCI 0	7 (19%)	2 (5%)	0.231^a^
CCI 1	7 (19%)	12 (30%)	
CCI 2	10 (27%)	17 (43%)	
CCI 3	7 (19%)	4 (10%)	
CCI 4	2 (5%)	3 (8%)	
CCI 5	1 (3%)	1 (3%)	
CCI 6	3 (8%)	1 (3%)	
Tumor characteristics			
T1	5 (14%)	7 (18%)	0.287^a^
T2	8 (22%)	12 (30%)	
T3	10 (27%)	4 (10%)	
T4	14 (38%)	17 (43%)	
N0	8 (22%)	6 (15%)	0.886^a^
N1	1 (3%)	1 (3%)	
N2	27 (73%)	32 (80%)	
N3	1 (3%)	1 (3%)	
Stage II	0 (0%)	1 (3%)	0.829
Stage III	3 (8%)	2 (5%)	
Stage IV	34 (92%)	37 (93%)	
p16 +	20 (54%)	26 (65%)	0.328^d^
p16-	17 (46%)	14 (35%)	
Dental status			
Edentulous at start RT	13 (35%)	9 (23%)	0.220^d^
Dentate at start RT	24 (65%)	31 (78%)	
Eichner index A at first assessment	7 (19%)	12 (30%)	0.427^d^
Eichner index B at first assessment	11 (30%)	8 (20%)	
Eichner index C at first assessment	19 (51%)	20 (50%)	
Eichner index A at start RT	4 (11%)	8 (20%)	0.547^a^
Eichner index B at start RT	13 (35%)	11 (28%)	
Eichner index C at start RT	20 (54%)	21 (53%)	
Decrease in Eichner index (ABC) due to tooth extractions prior to CRT/BRT	4 (11%)	5 (13%)	1.000^a^
No decrease in Eichner index (ABC) due to tooth extractions prior to CRT/BRT	33 (89%)	35 (88%)	
OU at first assessment; mean ± SD	3.5 ± 4.5	4.0 ± 4.7	0.642^b^
OU at start RT; mean ± SD	2.1 ± 3.6	3.2 ± 4.4	0.249^b^
Loss of OU due to tooth extractions prior to CRT/BRT			
Mean ± SD	1.4 ± 2.3	0.8 ± 1.8	
Median (IQR)	0.0 (3)	0.0 (1)	0.317^c^
Tooth extractions prior to CRT/BRT	20 (54%)	31 (78%)	**0.030**^**d**^
No tooth extractions prior to CRT/BRT	17 (46%)	9 (23%)	
Tooth extractions and/or additional interventions	23 (62%)	32 (80%)	0.083^d^
No tooth extractions and/or additional interventions	14 (38%)	8 (20%)	
Number of removed teeth; mean ± SD	3.4 ± 5.0	4.8 ± 6.1	0.289^b^
Treatment characteristics			
Primary CRT/BRT	35 (95%)	38 (95%)	1.000^a^
Postoperative CRT	2 (5%)	2 (5%)	
Cisplatin	27 (73%)	29 (73%)	0.963^d^
Cetuximab	10 (27%)	11 (28%)	
RT dose to contralateral submandibular gland (Gy); mean ± SD	48.1 ± 12.0*	49.7 ± 10.6*	0.529^b^
RT dose to contralateral parotid salivary gland (Gy); mean ± SD	24.2 ± 10.5	22.2 ± 7.1	0.345^b^
RT dose to superior PCM (Gy); mean ± SD	59.3 ± 11.6	59.3 ± 7.5	0.995^b^
RT dose to middle PCM (Gy); mean ± SD	59.8 ± 6.4	60.1 ± 7.1	0.870^b^
RT dose to inferior PCM (Gy); mean ± SD	49.4 ± 10.8	49.5 ± 8.4	0.939^b^
RT dose to oral cavity (Gy); mean ± SD	45.9 ± 11.0	45.2 ± 9.5	0.740^b^
RT dose to cricopharyngeal muscle (Gy); mean ± SD	44.5 ± 7.3	43.3 ± 6.5	0.433^b^
RT dose to cervical esophagus (Gy)			
Mean ± SD	41.5 ± 8.3	37.0 ± 11.1	
Median (IQR)	42.0 (8.0)	40.1 (17.7)	0.129^c^
TF during CRT/BRT (any duration)	24 (65%)	23 (58%)	0.508^d^
No TF	13 (35%)	17 (43%)	

*BMI* body mass index, *CCI* Charlson comorbidity index, *CRT/BRT* chemoradiotherapy or bioradiotherapy, *WHO PS* World Health Organization performance status, *p16* ± p16 positive/negative tumor as surrogate marker for human papilloma virus, *PCM* pharyngeal constrictor muscles, *RT* radiotherapy, *TF* tube feeding; TNM-classification, tumor (T), node (N), and metastasis (M) classification according to the 7th edition [34]

Bold values denote statistical significance at the *p* < 0.05 level

^a^Fisher’s exact test

^b^Independent *T*-test

^c^Mann-Whitney *U* test

^d^Chi^2^-test

^*^Two missing values due to a bilateral neck dissection

Univariable logistic regression analysis for significant weight loss during CRT/BRT revealed a potential associative value (*p* value < 0.10) for the factors BMI, tooth extractions, tooth extractions and/or additional interventions, and RT dose to the cervical esophagus (Table 3).

**Table 3:** Univariable and multivariable analysis of factors potentially contributing to a significant weight loss of > 5% during CRT/BRT and to TF dependency

	Significant weight loss of > 5% during CRT/BRT								TF dependency							
	Univariable analysis				Multivariable analysis*				Univariable analysis				Multivariable analysis*			
	OR	CI-95%		*p* Value	OR	CI-95%		*p* Value	OR	CI-95%		*p* Value	OR	CI-95%		*p* Value
		Lower	Upper			Lower	Upper			Lower	Upper			Lower	Upper	
Age	1.018	0.960	1.078	0.556					0.980	0.922	1.041	0.509				
Sex (male vs. female)	1.265	0.476	3.363	0.637					0.776	0.281	2.141	0.624				
Smoking	0.848	0.210	3.434	0.818					0.759	0.175	3.297	0.713				
Alcohol	1.968	0.781	4.956	0.151					0.982	0.386	2.501	0.970				
BMI	1.113	1.003	1.236	**0.044**	1.130	1.011	1.262	**0.031**	0.987	0.895	1.089	0.799				
Weight loss prior to CRT/BRT	0.941	0.823	1.075	0.370					1.187	1.001	1.407	**0.049**				
Dysphagia at start RT (CTCAE grade 2 vs. 0 or 1)	0.521	0.185	1.468	0.217					1.256	0.435	3.627	0.673				
WHO PS (1 or 2 vs. 0)	0.597	0.221	1.611	0.309					1.689	0.627	4.547	0.300				
CCI (≥ 4 vs. < 4)	0.738	0.205	2.659	0.642					0.732	0.202	2.650	0.634				
T3 or T4 vs. T0, T1 or T2	0.599	0.239	1.498	0.273					1.765	0.696	4.476	0.232				
N2 or N3 vs. N0 or N1	1.821	0.578	5.739	0.306					1.056	0.333	3.342	0.927				
p16 + vs. p16-	1.579	0.631	3.948	0.329					0.487	0.185	1.283	0.145				
Edentulous vs. dentate	0.536	0.197	1.462	0.223					0.892	0.325	2.447	0.825				
Decrease in Eichner Index (ABC) due to tooth extractions prior to CRT/BRT (binary)	1.179	0.291	4.771	0.818					0.465	0.114	1.894	0.285				
Tooth extractions (yes vs. no)	2.928	1.094	7.834	**0.032**	3.360	1.185	9.529	**0.023**	0.756	0.283	2.019	0.577				
Tooth extractions and additional interventions (yes vs. no)	2.435	0.877	6.756	0.087					0.484	0.165	1.425	0.188				
Number of removed teeth	1.047	0.961	1.140	0.291					0.995	0.917	1.080	0.909				
Loss of OU due to tooth extractions prior to CRT/BRT	0.867	0.687	1.095	0.232					1.125	0.877	1.445	0.354				
Cetuximab vs. cisplatin (ref)	1.024	0.375	2.795	0.963					0.355	0.127	0.995	**0.049**	0.226	0.070	0.731	**0.013**
RT dose to contralateral parotid gland	0.975	0.925	1.028	0.347					1.018	0.964	1.075	0.524				
RT dose to contralateral submandibular gland	1.013	0.973	1.056	0.523					1.048	1.001	1.096	**0.044**	1.067	1.013	1.124	**0.015**
RT dose to superior PCM	1.000	0.954	1.048	0.995					1.013	0.966	1.063	0.584				
RT dose to median PCM	1.006	0.941	1.075	0.868					1.040	0.970	1.115	0.272				
RT dose to inferior PCM	1.002	0.956	1.050	0.938					1.044	0.990	1.102	0.112				
RT dose to oral cavity	0.992	0.950	1.037	0.737					1.031	0.983	1.081	0.211				
RT dose to cricopharyngeus muscle	0.974	0.911	1.040	0.428					1.088	1.010	1.173	**0.026**				
RT dose to cervical esophagus	0.952	0.904	1.002	0.060					1.044	0.995	1.096	0.077				
TF use	0733	0.292	1.841	0.508												

*BMI* body mass index, *CCI* Charlson comorbidity index, *CRT/BRT* chemoradiotherapy or bioradiotherapy, *WHO PS* World Health Organization performance status, *OU* occlusal units, *PCM* pharyngeal constrictor muscles, *RT* radiotherapy, *TF* tube feeding

Boldface values denote statistical significance at the *p* < 0.05 level

^*^Step-backward analysis of all variables with *p* < 0.05 in univariable analysis

In multivariable step backward logistic regression analyses, tooth extractions prior to CRT/BRT and BMI at start of CRT/BRT remained as associative factors for weight loss > 5% during CRT/BRT, independent of weight loss prior to CRT/BRT, WHO PS, CCI, dental status at first assessment or at start CRT/BRT, number of occlusal units (OU), and number of removed teeth (Table 3). When evaluating the individual influence of potential associative factors, the associative value of extractions was reduced to a trend when corrected for alcohol use (*p* = 0.057).

Univariable logistic regression analysis for TF dependency during CRT/BRT revealed a potential associative value (*p* value < 0.10) for the following factors: weight loss prior to CRT/BRT, type of systemic therapy (cisplatin or cetuximab), RT dose to the contralateral submandibular gland, RT dose to the cricopharyngeal muscle, and RT dose to the cervical esophagus. None of the dental state parameters showed any significant associative value for TF dependency. In multivariable analysis, only a higher RT dose to the contralateral submandibular gland and type of systemic therapy (cisplatin) remained significant associative factors for the risk of TF dependency (Table 3).

## Discussion

The results of the current study showed that OPSCC patients who underwent tooth extraction(s) prior to IMRT intended to reduce the risk of ORN are more likely to experience significant weight loss of more than 5% during CRT/BRT. Interestingly, the number of teeth extracted and the number of functional units lost did not influence the degree of weight loss and the need for TF.

Few researchers studied the effect of dental status on weight loss or nutritional status in head and neck cancer patients. Thereby, uniform methods or widely accepted standardized protocols for dental status assessment are lacking. Despite the use of different study methods and dental status assessment methods, our results are in line with a study published in 2008 suggesting that dental condition, defined by the decayed, missing, and filled teeth index and the masticatory coefficient are risk factors for weight loss at the outset of management of head and neck cancer (HNC) [35]. Another study evaluated dental status by using the Eichner Index in a sample of 104 treatment-naïve HNC patients [36]. These authors reported that a reduced number of functional units was associated with the total nutrition impact symptoms score, but the absence of functional units was not necessarily an absolute impairment to achieve normal dietary intake. In our study, a reduced number of functional units were not associated with weight loss of more than five percent.

Limiting factors in previous studies were among others a mixture of tumor sites and limited information on possible associative factors. Also, no information was available on tooth loss in the context of pre-treatment tooth extractions or during oncological surgery, and data on weight loss during oncological therapy was underreported as well.

Research in the general population has shown a relationship between the number of natural teeth and weight loss. Having fewer teeth or being edentulous increased the risk of clinically relevant weight loss [37–40]. However, this concerns research among elderly people of at least 65 years of age, in which the dental status was examined and not the effect of tooth extractions as an intervention.

It remains unclear if the negative effect of tooth extractions on body weight is the result of a decrease in functional units or that it is the result of disrupting the existing masticatory system in its motor-sensory functionality and/or willingness to eat. Previous studies suggested that extractions, masticatory, and swallowing function are interrelated. The number of OU and having functional dentures were positively associated with masticatory performance in a prospective cohort study [11]. A retrospective single-center study in oral cancer patients showed that patients lacking OU had an increased risk for swallow impairment [41].

Therefore, an association between a deterioration of dental status, resulting in reduced masticatory performances, and weight loss seems conceivable.

Tooth extractions or functional units did not predict TF dependency. In a recent study in 450 LAHNSCC patients, nine associative values were added to a prediction model for the need for TF, including among others BMI and percentage weight change at baseline [42]. Since we only found the type of systemic therapy (cisplatin vs. cetuximab) and RT dose to the submandibular gland as independent TF predictors in the present study population, we have to assume that the study is underpowered and that these preliminary results should be interpreted with caution.

This is the first study addressing the impact of pre-CRT/BRT tooth extractions to reduce the risk of ORN, on weight loss. This weight loss is known to have a negative effect on treatment-related toxicity and oncological outcome. By evaluating the CRT/BRT trajectory, including neat weight reporting, a reliable retrospective assessment was possible. The addition of chemotherapy to RT as a radiosensitizer does not only enhance RT efficacy, but may also intensify side effects, including nausea, vomitus, mucositis, and weight loss [43, 44]. As a result, the percentage of patients who become TF-dependent during CRT/BRT could be higher than during RT as a single modality. Therefore, we focused on the vulnerable CRT/BRT group to answer our research question.

Despite the fact that the research was set up on the basis of strictly standardized usual care protocols, we have some limitations to address. The relatively small sample size impeded extensive subgroup stratification and multivariable corrections. The number of patients who were edentulous at baseline was relatively high. Edentulous patients may have had extractions (e.g., root tips or impacted wisdom teeth), but loss of a functional unit or decrease of the Eichner index is not possible. This may explain why extractions emerged as an associative factor for > 5% weight loss and the decline in OU and Eichner index did not reveal an association with weight loss. Although we were able to identify many factors associated with weight loss after tooth extractions, information on socio-economic and education status, factors associated with health perception, could not be retrieved from the electronic health records, as this information was not reported.

The patient’s financial and intellectual ability to modify their diet after tooth extractions may also have affected their capability to maintain weight, but accessing this privacy-sensitive data remains challenging. Following the procedure of tooth extraction, a reduced oral intake for approximately 1 or 2 weeks might lead to weight loss. Due to its retrospective character, we were not able to extract information on weight on the exact day of tooth extractions and on a standardized day after the procedure. However, a uniform moment of baseline measurements was defined, namely right before CRT/BRT initiation. Neither could we evaluate the effect of pain on oral intake since this was not reported in a standardized way and levels of treatment toxicity (mucositis, xerostomia) were not included in this study.

## Conclusion

Our study suggests that tooth extractions contribute to significant weight loss during treatment. Since bodyweight maintenance is important for completing planned oncological treatment and for supporting the recovery phase, further weight loss caused by tooth extractions should be minimized or avoided as much as possible. More careful consideration of teeth removal prior to CRT/BRT seems appropriate but demands close communication with the HNC team. As RT protocols and thus the doses to the tooth-bearing part of the jaws vary widely, interdisciplinary consultation with the radiation oncologist is highly recommended in order to reduce the risk of ORN due to potential oral sources of infection.

This study prompts further investigation into the adverse effects of tooth extractions and disruption of the masticatory system. That, along with the current improvements in RT techniques, may fuel the discussion to review and deescalate the current tooth extraction protocols aimed at reducing the risk of ORN.

## Data Availability

Not applicable.

## Abbreviations

BMI: Body mass index
BRT: Bioradiotherapy
CCI: Charlson comorbidity index
CRT: Chemoradiotherapy
CTCAE: Common terminology criteria for adverse events
HPV: Human papilloma virus
IMRT: Intensity-modulated radiotherapy
IQR: Inter quartile range
LAHNSCC: Locally advanced head and neck squamous cell carcinoma
MUMC +: Maastricht University Medical Center +
OPSCC: Oropharyngeal squamous cell carcinoma
ORN: Osteoradionecrosis
OU: Occlusal unit
PCM: Pharyngeal constrictor muscles
QoL: Quality of life
RT: Radiotherapy
SD: Standard deviation
TF: Tube feeding
WHO PS: World Health Organization performance status
